# Cooperative effects of SIRT1 and SIRT2 on APP acetylation

**DOI:** 10.1111/acel.13967

**Published:** 2023-08-21

**Authors:** Na Li, Ning Bai, Xiong Zhao, Rong Cheng, Xuan Wu, Bo Jiang, Xiaoman Li, Mingli Xue, Hongde Xu, Qiqiang Guo, Wendong Guo, Mengtao Ma, Sunrun Cao, Yanling Feng, Xiaoyu Song, Zhuo Wang, Xiaoyu Zhang, Yu Zou, Difei Wang, Hua Liu, Liu Cao

**Affiliations:** ^1^ Department of Gerontology and Geriatrics, Shengjing Hospital China Medical University Shenyang China; ^2^ The College of Basic Medical Science, Health Sciences Institute China Medical University Shenyang China; ^3^ Key Laboratory of Cell Biology of Ministry of Public Health, Key Laboratory of Medical Cell Biology of Ministry of Education, Liaoning Province Collaborative Innovation Center of Aging Related Disease Diagnosis and Treatment and Prevention China Medical University Shenyang China; ^4^ Department of Ophthalmology the First Affiliated Hospital of China Medical University Shenyang China; ^5^ Key Laboratory of Separation Science for Analytical Chemistry Dalian Institute of Chemical Physics, Chinese Academy of Sciences Dalian China; ^6^ Department of Histology and Embryology, The College of Basic Medical Science China Medical University Shenyang China; ^7^ Innovation Center of Aging‐Related Disease Diagnosis and Treatment and Prevention Jinzhou Medical University Jinzhou China

**Keywords:** aging, Alzheimer's disease, amyloidogenic processing, amyloid‐β, APP, deacetylation, SIRT1, SIRT2

## Abstract

Alzheimer's disease (AD) is an age‐related neurodegenerative disorder characterized by amyloid‐β (Aβ) deposition and neurofibrillary tangles. Although the NAD^+^‐dependent deacetylases SIRT1 and SIRT2 play pivotal roles in age‐related diseases, their cooperative effects in AD have not yet been elucidated. Here, we report that the SIRT2:SIRT1 ratio is elevated in the brains of aging mice and in the AD mouse models. In HT22 mouse hippocampal neuronal cells, Aβ challenge correlates with decreased SIRT1 expression, while SIRT2 expression is increased. Overexpression of SIRT1 prevents Aβ‐induced neurotoxicity. We find that SIRT1 impedes SIRT2‐mediated APP deacetylation by inhibiting the binding of SIRT2 to APP. Deletion of SIRT1 reduces APP recycling back to the cell surface and promotes APP transiting toward the endosome, thus contributing to the amyloidogenic processing of APP. Our findings define a mechanism for neuroprotection by SIRT1 through suppression of SIRT2 deacetylation, and provide a promising avenue for therapeutic intervention of AD.

## Graphical abstract

This study provides the new investigation of the cooperative effects of SIRT1 and SIRT2 on APP acetylation and neuronal survival. The SIRT2:SIRT1 ratio is elevated in the brains of aging mice and in the AD mouse models. Here, we show that in addition to inhibiting SIRT2 binding to APP, SIRT1 can act through a novel, catalytic‐independent mechanism to upregulate APP acetylation. The possible mechanism through which SIRT1 affects APP processing may be related to regulating APP trafficking and localization.

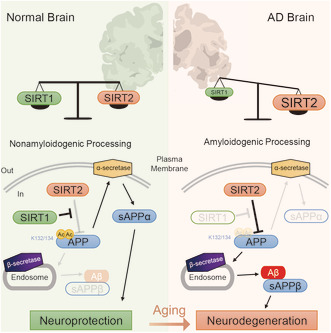

**Abbreviations**
5XFAD: transgenic mouse model of five familial Alzheimer's disease mutations
AD: Alzheimer's disease
ADAM10: A disintegrin and metalloproteinase 10
AFM: atomic force microscopy
APP: amyloid precursor protein
Aβ: amyloid β‐protein
BACE: beta‐site APP‐cleaving enzyme
co‐IP: co‐immunoprecipitation
Cx: cortex
EE: early endosomes
EEA: early endosome marker
ER: endoplasmic reticulum
GST: glutathione S‐transferase
HD: Huntington's disease
Hp: hippocampus
IPTG: isopropyl β‐D‐1‐thiogalactopyranoside
KO: knockout
LDH: lactate dehydrogenase
LE: late endosome
NAD: nicotinamide adenine dinucleotide
PBS: phosphate‐buffered saline
PD: Parkinson's disease
PS1: presenilin‐1
RARβ: retinoic acid receptor β
sAPPα: α secretase form of soluble amyloid precursor protein
sAPPβ: β secretase form of soluble amyloid precursor protein
SEM: standard error of the mean
Sir2: silent information regulator 2
WT: wild‐type

## 1 INTRODUCTION

Alzheimer's disease (AD) is an aging‐associated and progressive neurodegenerative disorder and has emerged as a major public health challenge for aging populations throughout the world (Livingston et al., 2017). AD patients suffer from impairments in cognition, learning, and memory (Braak & Braak, 1991; Markesbery, 2010). The characteristic pathological changes, including the deposition of amyloid‐β (Aβ) plaques and the accumulation of neurofibrillary tangles in brain tissue have been well‐established (Bloom, 2014; Hardy & Selkoe, 2002; Hardy & Higgins, 1992). However, the precise molecular mechanisms of the AD process remain unclear. Current research suggests that aging remains the main risk factor for developing AD, although it is also frequently associated with genetic and environmental factors.

Sirtuins, or silent information regulator 2 (Sir2) proteins, are nicotinamide adenine dinucleotide (NAD^+^)‐dependent deacetylases that play critical roles in delaying cellular senescence and extending lifespan by regulating various cellular processes (Carafa et al., 2016; Haigis & Sinclair, 2010; Jęśko et al., 2017). The mammalian sirtuins comprise seven homologs (SIRT1‐7), which have different enzymatic activities, sub‐cellular localization, and functional roles (Guarente, 2011; Michishita et al., 2005; Vassilopoulos et al., 2011). SIRT1, 6, and 7 are predominately located in the nucleus. SIRT2 is generally cytosolic, although it can be found transiently in the nucleus (North & Verdin, 2007a, 2007b). SIRT3, 4, and 5 are localized in the mitochondria (Verdin et al., 2010). With respect to enzymatic activity, SIRT1, 2, 3, 5, 6, and 7 possess deacetylase activity, SIRT4 and 6 show mono‐ADP‐ribosyl transferase activity, and SIRT5 also exerts demalonylase and desuccinylase activities (Donmez & Outeiro, 2013).

SIRT1 and SIRT2 are the most abundant sirtuins in the brain, and both are highly associated with neurodegenerative diseases including AD, Parkinson's disease (PD), and Huntington's disease (HD) (Ajami et al., 2017; Imai & Guarente, 2014). Several studies suggest that SIRT1 and SIRT2 promote opposite effects: although SIRT1 activation or overexpression is neuroprotective, SIRT2 upregulation is detrimental to neuronal cells (Chen et al., 2005; de Oliveira et al., 2017; Donmez & Guarente, 2010; Kim et al., 2007; Rizzi & Roriz‐Cruz, 2018). For example, overexpression of SIRT1 or pharmacological activation of SIRT1 appears to reduce Aβ production, oxidative stress, synapse loss, and cognitive impairment of AD mouse model (Godoy et al., 2014; Kim et al., 2007). Also, SIRT1 inhibits NF‐κB signaling by enhancing RelA/p65 deacetylation and protects against Aβ‐dependent neurodegeneration in microglia (Chen et al., 2005). In turn, SIRT2 inhibition is neuroprotective in AD, PD, and HD mouse models and delays the progression of neurodegeneration (Bai et al., 2022; Biella et al., 2016; Chen et al., 2015; Chopra et al., 2012; de Oliveira et al., 2017; Outeiro et al., 2007; Silva et al., 2017; Wang et al., 2020).

Amyloid precursor protein (APP) is a type I transmembrane glycoprotein that undergoes extensive post‐translational modification as it transits through the constitutive secretory pathway, including phosphorylation, sulfation, ubiquitination, sumoylation, N‐ glycosylation, and O‐glycosylation (Wang et al., 2017). Although acetylation signaling in AD mostly targets histone and Tau proteins (Lu et al., 2014; Min et al., 2015, 2018), much less is known about the acetylation of APP and how it impacts its function and processing. We recently reported that the acetylation of APP seems to promote non‐amyloidogenic processing of APP protein (Bai et al., 2022). While the neuroprotection of SIRT1 activation and SIRT2 inhibition have been reported, the common substrates of SIRT1 and SIRT2 and their cooperative effects in neurodegenerative disease have been less established. Thus, a better understanding of the association between SIRT1 and SIRT2 may improve the therapeutic interventions against AD.

In this study, we provide evidence that SIRT1 physically interacts with APP. Overexpression of SIRT1 impedes SIRT2‐mediated APP deacetylation. In addition, SIRT1 overexpression protects SH‐SY5Y cells from Aβ‐induced defects. Importantly, SIRT1 deletion promotes APP retention at the endosome and reduces APP at the cell surface. Our study advances our understanding of the cooperation between SIRT1 and SIRT2 on APP acetylation, which may benefit the therapeutic strategy against AD.

## 2 RESULTS

### 2.1 SIRT2:SIRT1 ratio increases in the brains of aging and AD mice

The expression levels of SIRT1 and SIRT2 differ considerably during aging and the development of AD. A reduction of SIRT1 activity or level has been observed in both aged rat brain and old people, and in the brain of AD patients compared to young groups (Braidy et al., 2015; Quintas et al., 2012; Wongchitrat et al., 2019). The level of SIRT1 in postmortem human brain samples from AD patients correlates with the Braak stage and disease duration (Cao et al., 2018; Lutz et al., 2014). Given these observations, we asked whether the SITR2:SIRT1 ratio also changed in our mouse model.

**FIGURE 1:**
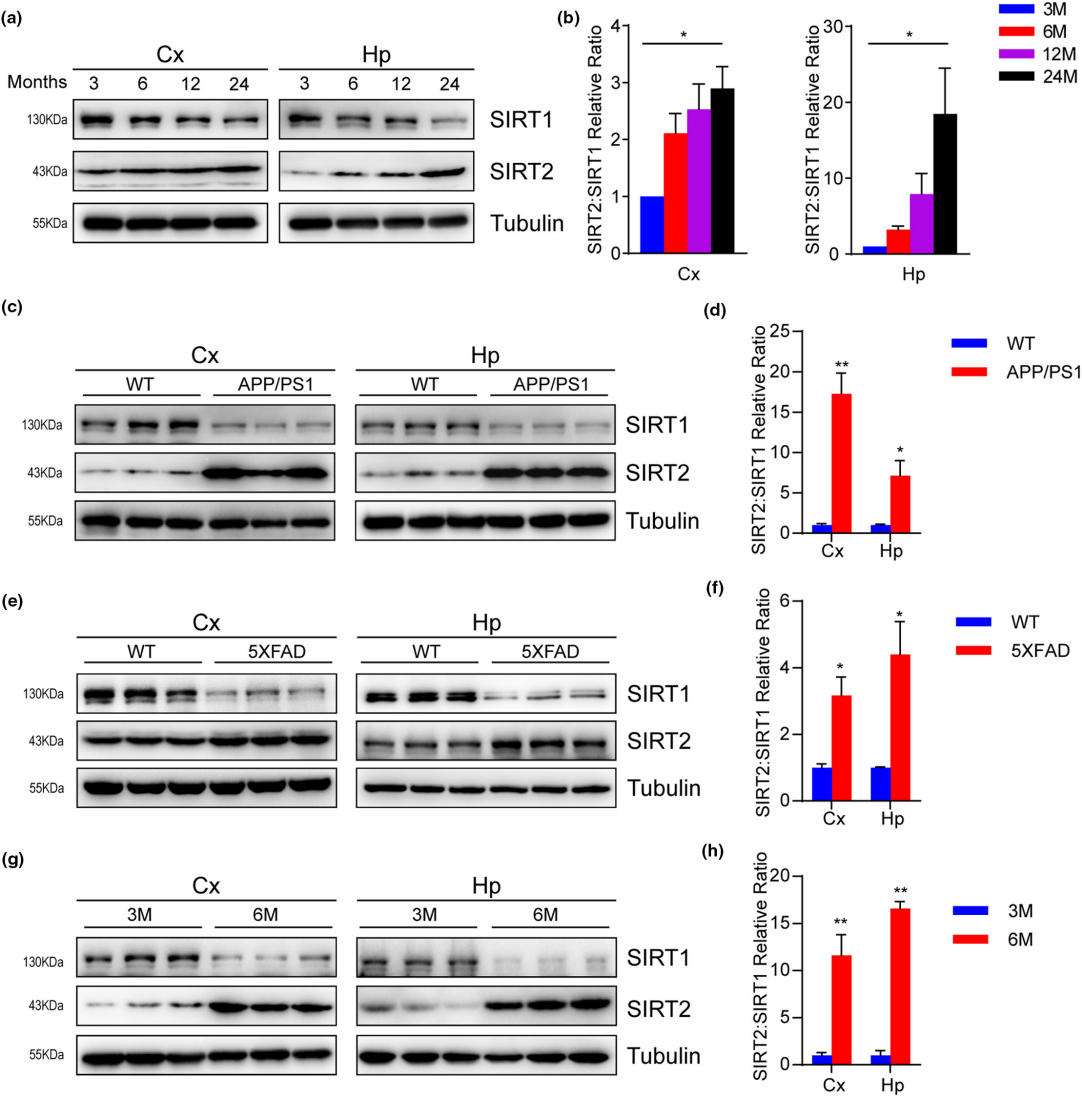
SIRT2:SIRT1 ratio increases in the brains of aging and AD mice. (a, b) Western blotting and quantification of the protein expression levels of SIRT1 and SIRT2 from whole brains isolated from 3, 6, 12 and 24‐month‐old C57BL/6 WT mice (*n* = 3 per group). (c, d) The protein level of SIRT1 and SIRT2 in the brains of APP/PS1 mice compared to WT mice. Quantification of the SIRT2: SIRT1 ratio (*n* = 3). (e, f) The protein level of SIRT1 and SIRT2 in the brains of 9‐month‐old 5XFAD mice compared to WT mice. Quantification of the SIRT2: SIRT1 ratio (*n* = 3). (g, h) Western blotting and quantification of the protein expression level of SIRT1 and SIRT2 in the brains of 3‐ and 6‐month‐old APP/PS1 mice (*n* = 3). Data are means ± SEM. Asterisk depicts *p* values (*p < 0.05, **p < 0.01) as observed by one‐way ANOVA (b) or Student's *t* test (d, f, h), as applicable.

We first evaluated the levels of SIRT1 and SIRT2 during aging. Brain extracts isolated from mice at 3, 6, 12, and 24 months of age were subjected to Western blotting analysis. SIRT2 expression increased with aging, whereas SIRT1 expression decreased gradually (Figure 1a). Thus, the SIRT2:SIRT1 ratio gradually increased with age (Figure 1b). To extend these observations to the AD context, we applied two AD animal models, for example, APP/PS1 mice (Casas et al., 2004) and 5XFAD mice (Oakley et al., 2006). Consistent with a previous report of an increased SIRT2:SIRT1 ratio in postmortem AD individuals (Theendakara et al., 2013), we observed the SIRT2:SIRT1 ratio was higher both in the cortex and hippocampus of APP/PS1 mice compared to their littermates (wild‐type, WT) at the age of 6 months (Figure 1c,d). In addition, 5XFAD mice also showed an increased SIRT2:SIRT1 ratio at the age of 9 months compared to their WT littermates (Figure 1e,f). Next, we compared the expression of SIRT1 and SIRT2 in 3 and 6‐month‐old APP/PS1 mice. We also observed that the expression of SIRT1 decreased, the expression of SIRT2 increased, and the ratio of SIRT2 to SIRT1 also increased (Figure 1g,h). Consistently, immunostaining revealed a decrease in SIRT1 expression and an increase in SIRT2 expression in the frontal cortex and hippocampus of APP/PS1 mice (Figure S1a). Taken together, our data suggest that SIRT1 and SIRT2 may be involved in aging and neurodegeneration.

### 2.2 SIRT1 and SIRT2 have opposite effects on cell survival under Aβ42 challenge

**FIGURE 2:**
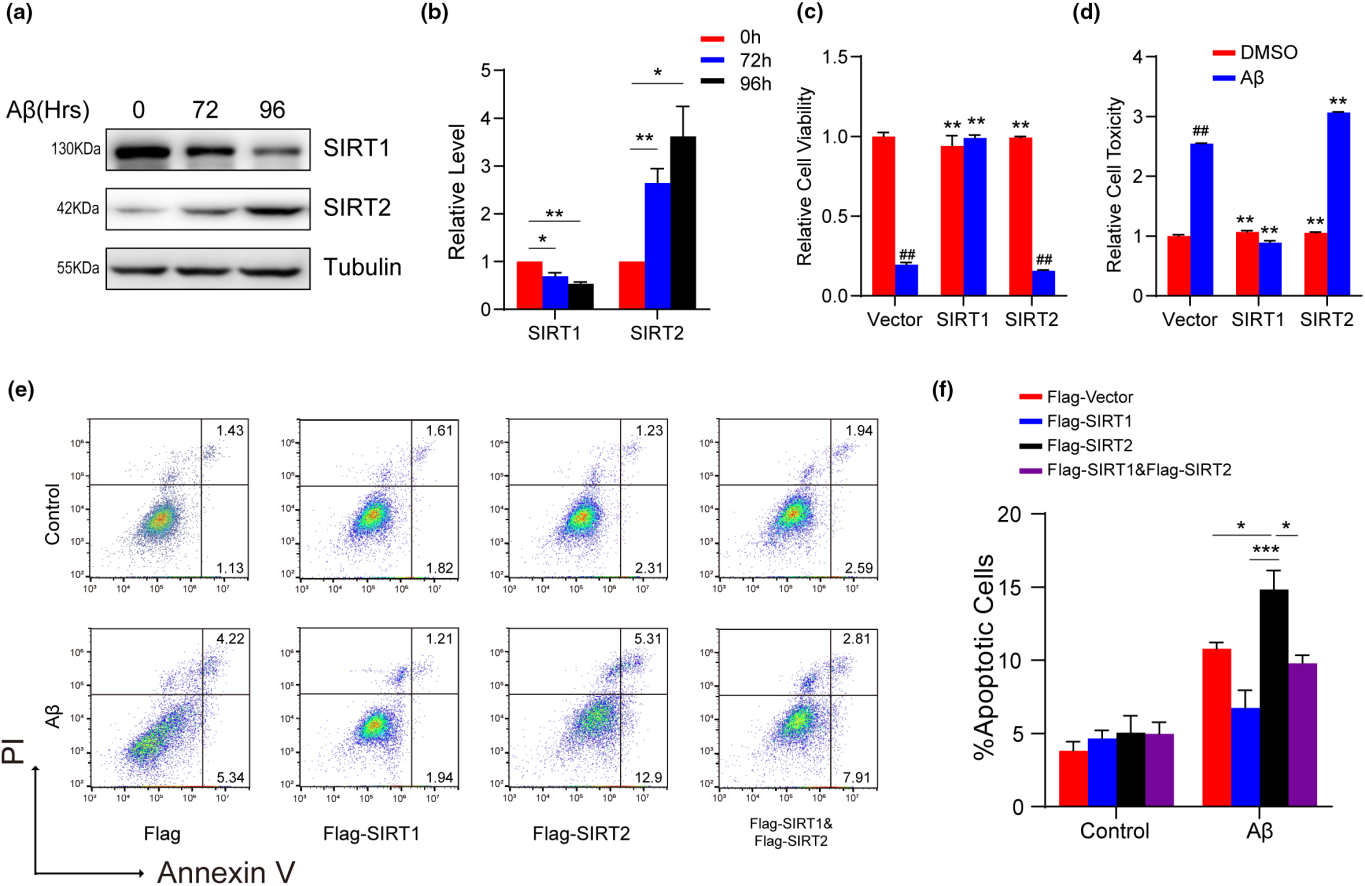
SIRT1 and SIRT2 have opposite effects on cell survival under Aβ42 challenge. (a, b) Western blotting and quantification of the protein expression level of SIRT1 and SIRT2 upon Aβ42 stimulus (10 μΜ) in HT22 cells (*n* = 3). (c) Cell viability was measured by the CCK8 assay. (d) Lactate dehydrogenase levels (LDH) were measured in the supernatants of SH‐SY5Y cells (*n* = 4). ** indicates *p* < 0.01 compared with empty vector + Aβ group; ## indicates *p* < 0.01 compared with empty vector + DMSO group. (e, f) Fluorescence‐activated cell sorting (FACS) and statistical analysis showing apoptosis of indicated SH‐SY5Y cells (*n* = 3). Data are means ± SEM. Asterisk depicts *p* values (*p < 0.05, **p < 0.01) as observed by Student's *t* test (b–d) or one‐way ANOVA with Tukey's multiple comparison test (f), as applicable.

We next asked if treatment of HT22 mouse hippocampal neuronal cells with amyloid‐beta (Aβ), the major contributor to AD, affects the protein expression level of SIRT1 and SIRT2 over several time points. We prepared Aβ42 oligomers and confirmed them by atomic force microscopy (AFM; Wang et al., 2023; Figure S2a). We found that Aβ stimulation (10 μM) markedly reduced SIRT1 protein and upregulated SIRT2 protein at 72 and 96 h (Figure S2b,c; Figure 2a,b). Thus, similar to the in vivo experiment results, Aβ also increased the SIRT2:SIRT1 ratio in a time‐dependent manner.

We then assessed how the changes in SIRT1 and SIRT2 may differentially impact the Aβ‐induced defects. SH‐SY5Y neuroblastoma cells were transfected with empty vector, Flag‐tagged SIRT1 or SIRT2 and then treated with Aβ42 oligomers (10 μM, 70 h). Aβ markedly reduced cell survival in the cells transfected with empty vector, but SIRT1 overexpression substantially counteracted the Aβ‐induced cell damage (Figure 2c). By contrast, SIRT2 overexpression reduced the viability of Aβ42‐treated cells compared to untreated cells (Figure 2c). Moreover, the Aβ stimulus seemed to correlate with increased cytotoxicity in empty vector and SIRT2 overexpressed cells, as indicated by measuring the lactate dehydrogenase (LDH) levels in cell culture supernatants (Figure 2d). On the other hand, Aβ‐induced cell damage was significantly rescued by SIRT1 overexpression (Figure 2d). Moreover, SIRT1 expression attenuated Aβ‐induced apoptosis, whereas the SIRT2‐transfected cells were more sensitive to Aβ‐induced neurotoxicity (Figure 2e,f). SIRT1 can alleviate cell apoptosis caused by overexpression of SIRT2 in response to Aβ challenge. Together, these data suggest that while SIRT1 protects against neuronal death, SIRT2 promotes neurotoxicity, SIRT1 and SIRT2 have opposite effects on cell survival under Aβ42 challenge.

### 2.3 SIRT1 competes with SIRT2 for binding to APP

**FIGURE 3:**
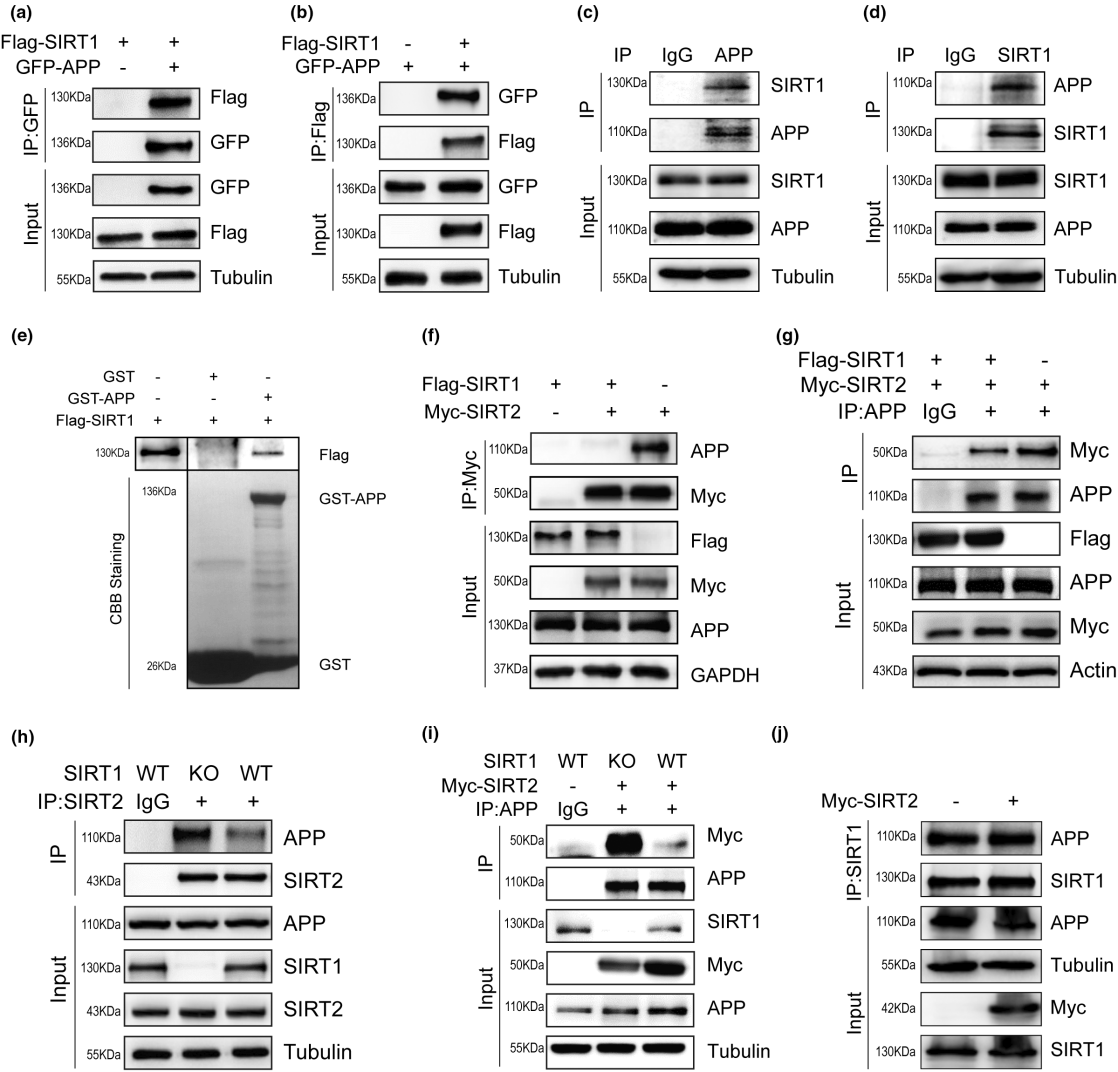
SIRT1 competes with SIRT2 for binding to APP. (a, b) Co‐IP analysis of the interaction of exogenous SIRT1 and APP in HEK293T cells. (c, d) Co‐IP analysis of the interaction of endogenous SIRT1 and APP in mouse brain. IgG, immunoglobulin G. (e) GST pull‐down assay of Flag‐SIRT1 fusion proteins with bacterially expressed GST‐APP fragments. CBB staining shows the expression of GST and GST‐APP fragments. (f, g) Co‐IP of Myc‐tagged SIRT2 and APP with or without Flag‐tagged SIRT1 in HEK293T cells. (h, i) Co‐IP of SIRT2 and APP in WT or SIRT1 KO‐ HEK293 cells. (j) Co‐IP of SIRT1 and APP in the presence or absence of Myc‐tagged SIRT2 in HEK293T cells. (*n* = 3 for each experiment).

Our previous study revealed that SIRT2 binds to and deacetylates APP at lysine 132 and 134 (Bai et al., 2022). In order to explore whether SIRT1 is also a binding partner of the APP protein, we first conducted co‐immunoprecipitation (co‐IP) assays in human embryonic kidney (HEK) 293 T cells. We observed a clear interaction between APP and SIRT1 (Figure 3a,b). We then looked for a physiological association by performing IP of endogenous SIRT1 or APP proteins from mouse brain lysates. APP co‐IPed with SIRT1, and vice versa (Figure 3c,d). We further confirmed the direct interaction between APP and SIRT1 by glutathione S‐transferase (GST) pull‐down assays (Figure 3e).

Next, we tested the effect of SIRT1 on the interaction between SIRT2 and APP and found that it was significantly weakened in the presence of SIRT1 (Figure 3f,g). Conversely, the binding of SIRT2 to APP was enhanced when SIRT1 was absent (Figure 3h,i). Furthermore, SIRT2 had no effect on the interaction between SIRT1 and APP (Figure 3j). These findings suggest that the presence of SIRT1 acts as a context‐dependent regulatory factor affecting the interaction between SIRT2 and APP.

### 2.4 SIRT1 impedes SIRT2‐mediated APP deacetylation

**FIGURE 4:**
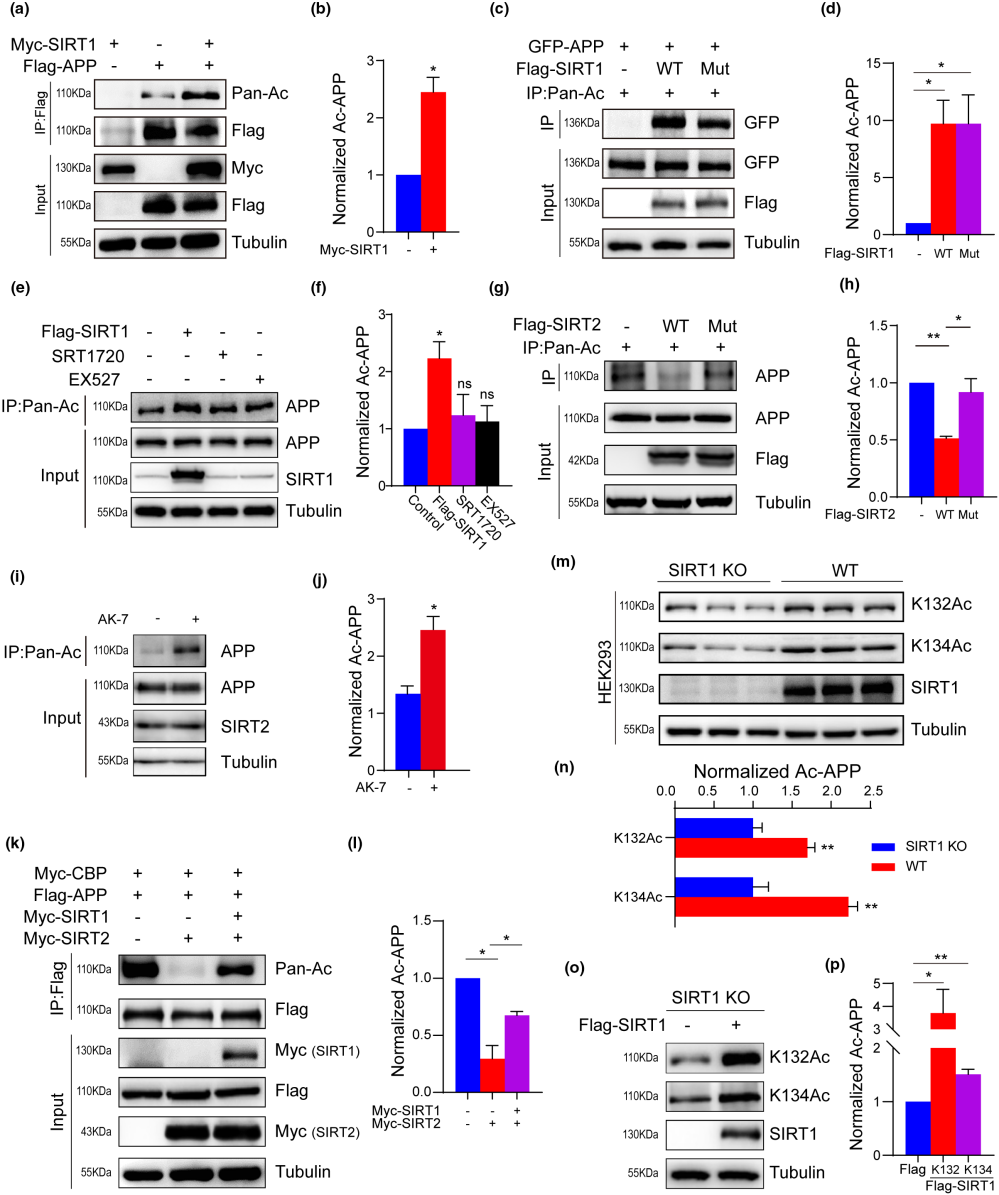
SIRT1 impedes SIRT2‐mediated APP deacetylation. (a, b) Acetylation of APP significantly increased with SIRT1 overexpression in HEK293T cells. Quantification of the acetylation of APP in the left panel. (c, d) Acetylation of APP increased with wild‐type and mutant SIRT1 overexpression. Quantification of the acetylation of APP in the left panel. (e, f) HEK 293 cells were transfected with Flag‐SIRT1 or treated with SIRT1 agonist SRT1720 (10 μM, 24 h) and SIRT1 inhibitor EX527 (10 μM, 24 h). APP acetylation was quantified. (g, h) Acetylation and quantification of APP with wild‐type and mutant SIRT2 overexpression. (i, j) HEK 293 cells were treated with SIRT2 inhibitor AK7 (10 μM, 24 h). APP acetylation was quantified. (k, l) IP and quantitative analysis showing the effect of SIRT1 on the acetylation of APP proteins in HEK293T cells transfected with or without Myc‐SIRT2. (m, n) Western blot and quantitative analysis of APP acetylation at K132 or K134 in HEK293‐SIRT1 KO and WT cells. (o, p) Acetylation of APP at K132 or K134 significantly increased with SIRT1 overexpression in HEK293‐SIRT1 KO cells. Quantification of the acetylation of APP in the left panel. The data are means ± SEM, *n* = 3 for each experiment. Asterisk depicts *p* values (*p < 0.05, **p < 0.01) as observed by Student's *t* test (b, d, f, h, j, l, n, p), as applicable.

Considering that both SIRT1 and SIRT2 can bind APP, we examined whether they cooperate with each other to regulate APP protein acetylation. To test this, we co‐transfected Flag‐tagged APP with or without Myc‐tagged SIRT1 into HEK293T cells. SIRT1 overexpression significantly increased the overall acetylation of APP (Figure 4a,b), so did the catalytically inactive H363Y mutant SIRT1 (Vaziri et al., 2001; Figure 4c,d), suggesting that the catalytic activity of SIRT1 is not essential for acetylation of APP. Moreover, we treated cells using SIRT1 agonist SRT1720 and inhibitor EX527, then evaluated the acetylation of APP. As shown in Figure 4e,f, SRT1720 and EX527 had no effect on APP acetylation levels, providing further evidence that SIRT1 enhances APP acetylation in a catalytic‐independent manner. Although expression of wild‐type SIRT2, not the catalytically inactive H187Y mutant (North et al., 2003; North & Verdin, 2007a, 2007b), led to a deacetylation of APP (Figure 4g,h). On the contrary, SIRT2 inhibitor AK7 could increase the acetylation of APP (Figure 4i,j).

We then asked whether the SIRT2‐mediated reduction in APP acetylation could be reversed by SIRT1 overexpression. HEK293T expressing Flag‐APP and Myc‐SIRT2 were transfected with or without Myc‐tagged SIRT1. SIRT2 significantly decreased APP acetylation; however, this effect was prevented by the overexpression of SIRT1 (Figure 4k,l). Moreover, knockout of SIRT1 decreased APP acetylation on K132 and K134 residues that are regulated by SIRT2 (Figure 4m,n), and this reduction was reversed by SIRT1 overexpression in SIRT1 knockout HEK293 cells (Figure 4o,p). These results indicate that SIRT1 attenuates SIRT2‐mediated APP deacetylation and increases the acetylation level of APP through inhibiting the binding of SIRT2 to APP. Taken together, our findings suggest that the homeostasis of APP acetylation is co‐regulated by SIRT1 and SIRT2 proteins.

### 2.5 SIRT1 promotes non‐amyloidogenic processing of APP by altering its localization

**FIGURE 5:**
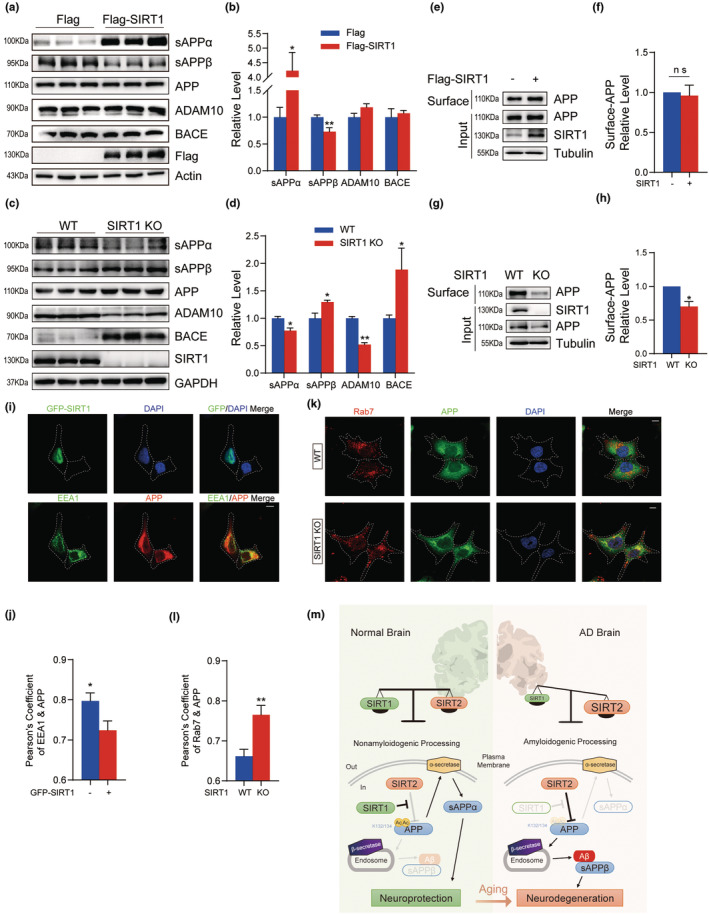
SIRT1 Promotes non‐amyloidogenic processing of APP by altering its localization. (a, b) Western blot analysis and quantification of the expression levels of sAPPα, sAPPβ, APP, SIRT1, ADAM10, BACE, and Actin in HEK293 cells transfected with Flag or Flag‐SIRT1 (*n* = 3). (c, d) Western blot analysis and quantification of the expression levels of sAPPα, sAPPβ, APP, SIRT1, ADAM10, BACE, and GAPDH in HEK293 or HEK293‐SIRT1 KO cells (*n* = 3). (e, f) Streptavidin pull‐down assay and quantification of surface (biotinylated) APP in HEK293 cells transfected with indicated vectors (*n* = 4). (g, h) Streptavidin pull‐down assay and quantification of surface APP in HEK293 cells or HEK293‐SIRT1 KO cells (*n* = 4). (i) IF of HEK293 cells using the indicated antibodies. Dashed lines indicate the left cell transfected with GFP‐SIRT1, but the right cell not transfected with GFP‐SIRT1 (Scale bar = 10 μm). (j) Quantitative analysis of co‐localization of APP with EEA1 in E (*n* = 8 fields of vision). (k) IF of HEK293 cells or HEK293‐SIRT1 KO cells using the indicated antibodies. Dashed lines indicate the cell profile (Scale bar = 10 μm). (l) Quantitative analysis of co‐localization of APP with Rab7 in e (*n* = 8 fields of vision). (m) Proposed model for cooperative effects of SIRT1 and SIRT2 on regulating APP processing pathway via targeting APP acetylation. Data are means ± SEM. Asterisk depicts *p* values (*p < 0.05, **p < 0.01) as observed by Student's *t* test (b, d, f, h, j, l), as applicable.

As previously reported, SIRT1 regulates the balance between non‐amyloidogenic and amyloidogenic processing of APP through multiple targets (Guo et al., 2016; Qin et al., 2006; Wang & Tian, 2012). We next examined the processing products of APP in HEK293 cells. In contrast to increased sAPPα levels with SIRT1 overexpression in WT cells (Figure 5a,b), we observed a reduction of sAPPα in SIRT1‐deficeint cells (Figure 5c,d). Furthermore, SIRT1 also decreased the production of sAPPβ but had no significant effect on the total levels of APP, ADAM10, and BACE (Figure 5a,b). Deletion of SIRT1 decreased the production of sAPPα and ADAM10 levels but increased the production of sAPPβ and BACE (Figure 5c,d).

APP processing is regulated by its trafficking and localization (Zhang et al., 2011). To assess whether SIRT1 affects APP endocytosis, we performed a cell‐surface biotinylation assay. We found that SIRT1 overexpression had no effect on the amount of biotinylated APP at the cell surface (Figure 5e,f), whereas SIRT1 deficiency significantly decreased surface APP (Figure 5g,h). To determine if SIRT1 could modulate APP localization in endosome compartment, where amyloidogenic APP processing mainly occurs, we used a confocal immunofluorescence assay. SIRT1 overexpression significantly decreased the APP retained in the early endosome (EE; Figure 5i,j), while overexpression of SIRT2 could not change APP localization in EE (Figure S3a,b). On the contrary, SIRT1 deletion increased the localization of APP in these compartments (Figure 5k,l), and consequently triggered amyloidogenic APP processing. Thus, our data indicate that SIRT1 overexpression decreases the amyloidogenic proteolytic pathway by reducing APP in the endosome. On the contrary, SIRT1 deficiency decreases the non‐amyloidogenic pathway through reducing APP residing at the cell surface and increasing APP retention in the late endosome (Figure 5m).

## 3 DISCUSSION

Although the role of the APP protein in AD has been extensively studied, the critical function of APP acetylation in AD is just beginning to emerge. In this work, we show that SIRT1 and SIRT2 cooperatively regulate the balance between APP acetylation and deacetylation. SIRT2 deacetylates APP, while SIRT1 promotes APP protein acetylation by inhibiting SIRT2 binding to APP. In the aging and AD contexts, the expression of protective SIRT1 decreases, while the level of detrimental SIRT2 increases. Notably, this change may result in a reduction of APP acetylation, leading to the amyloidogenic processing of APP (Figure 5m).

SIRT1 and SIRT2 shift the balance of APP processing in opposing ways. For example, through deacetylating the Retinoic acid receptor β (RARβ), SIRT1 activates α‐secretase ADAM10 transcription, leading to upregulated non‐amyloidogenic processing of APP (Lee et al., 2014; Tippmann et al., 2009). By inhibiting NF‐κB signaling, SIRT1 downregulates the expression of the β‐secretase β‐site APP‐cleaving enzyme 1 (BACE1), and consequently reduces the amyloidogenic pathway (Gao et al., 2015; Guo et al., 2016). Moreover, SIRT1 also reduces BACE1 transcription through PGC1‐α and PPARγ deacetylation, thereby reducing Aβ production (Wang & Tian, 2012). Surprisingly, in this study, overexpression of SIRT1 did not change the expression of ADAM10 and BACE1; however, we observed that the loss of SIRT1 caused a reduction in ADAM10 and an increase in BACE1. On the other hand, SIRT2 inhibition reduced APP amyloidogenic processing through downregulating BACE1 and increased non‐amyloidogenic APP processing through increasing APP acetylation and reducing Aβ generation in APP/PS1 mice (Bai et al., 2022; Wang et al., 2020).

The pathogenesis of many neurodegenerative diseases has been currently hypothesized to be neuroinflammation, oxidative stress, and apoptosis. Emerging evidence suggests important roles for sirtuins deacetylase in regulation of neuronal survival and plasticity, redox balance, and energy homeostasis by altering protein acetylation status (Calabrese et al., 2010; Khan et al., 2021). Given the relationship between vitagene networks and their possible biological relevance in defense mechanisms against oxidative stress‐driven degenerative diseases, the therapeutic potential of these targets in models of neurodegenerative diseases can be expedited (Calabrese et al., 2007, 2009, 2010; Siracusa et al., 2020).

Here, we have described a novel pathway by which SIRT1 promotes non‐amyloidogenic cleavage of APP in vitro. Nascent APP generated in the endoplasmic reticulum (ER) undergoes post‐translational modification and traffics through the constitutive secretory pathway (Haass et al., 2012). When it reaches the plasma membrane where α‐secretase is enriched, APP is mainly cleaved in the non‐amyloidogenic processing pathway, leading to the production of neuroprotective sAPPα. APP may also be internalized into the late endosome (LE), where β‐secretase is mostly located, thereby initiating the amyloidogenic APP processing (Wilkins & Swerdlow, 2017). Increasing evidence suggests that the important role of post‐translational modifications, especially phosphorylation, is in regulating APP metabolism and Aβ biosynthesis (Lee et al., 2003; Rebelo et al., 2007; Wang et al., 2017; Zhang et al., 2019). We found SIRT1 may regulate APP trafficking and localization. Deletion of SIRT1 reduced APP recycling back to the cell surface and promoted APP transiting towards acidic late endocytic organelles (LE). In contrast, overexpression of SIRT1 decreased the APP retained in the early endosomes (EE). Surprisingly, SIRT1 overexpression did not lead to an increase in APP residing on the cell surface. Another possible mechanism by which SIRT1 increases non‐amyloidogenic processing is through increasing APP acetylation, like SIRT2 inhibition. Our findings draw attention to a new mechanism by which SIRT1 effects APP modification and processing.

Aβ was found to significantly suppress the mRNA expression and protein level of SIRT1 (An et al., 2022). Here, we found that Aβ not only decreased SIRT1 but also increased SIRT2 expression, which is consistent with the increased ratio of SIRT2:SIRT1 observed in our mouse models. In addition, overexpression of SIRT1, as expected, could counteract the neurotoxic effects of Aβ in SIRT2‐transfected cells. This further indicates that the reduced SIRT1 and increased SIRT2 levels are harmful during the aging and AD processes. Consequently, on the one hand, the neuroprotective effect of SIRT1 itself is reduced, while on the other hand, its ability to counteract the detrimental effects of SIRT2 is decreased, which leads to the occurrence of aging and neurodegeneration.

In conclusion, our study provides the first investigation of the cooperative effects of SIRT1 and SIRT2 on APP acetylation and neuronal survival. We show that in addition to inhibiting SIRT2 binding to APP, SIRT1 can act through a novel, catalytic‐independent mechanism to upregulate APP acetylation. The possible mechanism through which SIRT1 affects APP processing may be related to regulating APP trafficking and localization. Thus, therapies targeting the cooperation of SIRT1 and SIRT2‐mediated APP acetylation could provide a potential candidate treatment for AD.

## 4 METHODS

### 4.1 Animals

The male and female APP/PS1 (B6C3‐Tg [APPswe, PSEN1dE9] 85Dbo/Mmjax) transgenic mice were obtained from the Jackson Laboratory. 5XFAD transgenic mice were obtained from (Beijing HFK Bioscience Co.). Wild‐type littermates were used as controls. All animals were housed in a controlled environment and had free access to a standard diet and distilled water. All animal experimental procedures were approved by the Laboratory of Animal Ethical Committee of China Medical University (animal protocol # CMU2018342).

### 4.2 Antibodies and reagents

Rabbit anti‐SIRT1 (1:1000, Sigma, 07131), rabbit anti‐APP (1:5000, Abcam, ab32136), mouse anti‐APP (1:5000, Millipore, MAB348), rabbit anti‐SIRT2 (1:1000, Sigma, S8447), rabbit anti‐SIRT2 (1:1000, Abcam, ab211033), mouse anti‐SIRT2 (1:1000, ZEN Bioscience, 200474), rabbit anti‐Aβ (1:1000, CST, 8243), rabbit anti‐A disintegrin and metalloproteinase 10 (ADAM10, 1:1000, CST, 14194), rabbit anti‐BACE (1:1000, CST, 5606), rabbit anti‐Presenilin 1 (PS1, 1:1000, CST, 5643), mouse anti‐sAPPα (1:1000, Immuno‐Biological Laboratories (IBL), 11088), mouse anti‐sAPPβ‐sw (1:1000, IBL, 10321), rabbit anti‐acetylated‐lysine (1:1000, CST, 9441), rabbit anti‐EEA1 (1:100–1:1000, CST, 3288), mouse anti‐Rab7 (1:100, Santa Cruz, sc‐376362), mouse anti‐GFP (1:1000, Immunoway, YM3124), rabbit anti‐GAPDH (1:5000, proteintech, 60004‐1‐Ig), and Flag (1:1000, SG4110‐16, Shanghai Genomics Technology), Myc (1:1000, SG4110‐18, Shanghai Genomics Technology), rabbit AAP‐K132‐AC, AAP‐K134‐AC (Bai et al., 2022). SRT1720 was purchased from Selleck (S1129). EX527 was purchased from Selleck (S1541). Human Synthetic Amyloid‐β (1–42) was purchased from ChinaPeptides.

### 4.3 Cell culture

HEK293T, HEK293, and SH‐SY5Y cells were purchased from Cell Bank in the Chinese Academy of Sciences Shanghai. The HT22 mouse hippocampal neuronal cells were a gift from Prof. Lihong Jia (China Medical University). HEK293T, HEK293, and HT22 cells were cultured in high‐glucose Dulbecco's modified Eagle's medium (DMEM) supplemented with fetal bovine serum (FBS; 10%, CLARK, Australia), penicillin (100 U), and streptomycin (100 μg/mL) in 5% CO_2_ at 37°C. SH‐SY5Y cells were cultured in high‐glucose DMEM/F12 (Genview, GF3105) supplemented with 10% FBS, penicillin (100 U), and streptomycin (100 μg/mL) in 5% CO_2_ at 37°C.

### 4.4 Brain tissue preparation

For morphological analyses, animals were deeply anesthetized with sodium pentobarbital (50 mg/kg, i.p.) and fixed by transcardiac perfusion of 4% paraformaldehyde in 0.1 M phosphate‐buffered saline (pH 7.4, PBS). The removed brain was further fixed by immersion in the same fixative overnight. After gradient sucrose dehydration, brains were embedded in the OCT compound and stored at −80°C before freezing and cutting. For biochemical and molecular biologic analyses, whole brains were dissected from the mice sacrificed by decapitation, the cortex and hippocampus were then dissected and immediately submerged in ice‐cold PBS. Then, the dissected cortex and the hippocampus were snap‐frozen in liquid nitrogen and stored at −80°C until use.

### 4.5 Immunohistochemistry

For immunohistochemistry staining, paraffin‐embedded brain sections (4 μm) were deparaffinized and rehydrated. Antigen retrieval was performed by boiling for 90 s in sodium citrate buffer (10 mM, pH 6.0) using a pressure cooker. Sections were stained with a immunostaining kit (KIT‐9720, MXB Biotechnologies). Tissue sections were incubated with antibodies for SIRT1 (1:250, Sigma, 07131), SIRT2 (1:200, ZEN Bioscience, 200474) overnight at 4°C. Antibody binding was detected using HRP‐conjugated anti‐rabbit or mouse secondary antibody and visualized using DAB Peroxidase Substrate kit (DAB‐0031, MXB Biotechnologies). The images were acquired using a microscope (Nikon, DS‐Fi2).

### 4.6 Immunocytochemistry

For immunocytochemistry analysis, HEK293 cells were grown on coverslips and were fixed with 4% paraformaldehyde for 10 min at room temperature and subsequently blocked with 0.1% Triton X‐100/10% goat serum for 30 min at room temperature. Fixed cells were incubated overnight at 4°C with the corresponding primary antibodies: rabbit anti‐APP (Millipore, MAB348, 1:200 dilution), rabbit anti‐EEA1 (CST, 3288, 1:100 dilution), rabbit anti‐APP (Abcam, ab32136, 1:200 dilution), mouse anti‐Rab7 (Santa Cruz, sc‐376362, 1:100 dilution). Coverslips were then washed in phosphate‐buffered saline (PBS) and stained for 60 min with Alexa Fluor 488‐, Alexa Fluor 568‐, and Alexa Fluor 647‐conjugated secondary antibodies (Invitrogen; 1:400). Coverslips were washed again with PBS and mounted with DAPI (Sigma, 32670). Imaging was performed on GE Deltavision OMX SR using a Plan Apo N 60× 1.42 Oil. Image reconstruction and processing were performed using SoftWoRx 8.0.0 software.

### 4.7 Western blot and IP analyses

Western blotting was performed as previously (Bai et al., 2022) described. Cells or tissues were lysed with modified lysis buffer (150 mM NaCl, 1 mM NaF, 50 mM Tris–HCl (pH 7.6), 1 mM EDTA, 1 mM Na_3_VO_4_, 1% NP‐40, 1 mM MgCl_2_, 1 mM DTT, 1 mM NaF, 0.25% sodium deoxycholate) containing protease inhibitor cocktail (Sigma) and 1 mM phenylmethyl sulfonyl fluoride (PMSF) on ice and then subjected to a BCA assay (Pierce). For acetylation IP, 5 μM TSA and 20 mM NAM were added to the cell lysis buffer (137 mM NaCl, 10 mM NaF, 50 mM Tris–HCl (pH 7.6), 1 mM EDTA, 0.1 mM Na_3_VO_4_, 10% glycerol, 1% NP‐40, and 1 mM PMSF). Cell or tissue lysates were either incubated with anti‐Flag beads at 4°C overnight or incubated with the appropriate antibodies for 3 h at 4°C and subsequently with protein A/G‐Sepharose beads overnight at 4°C. Thereafter, the protein‐antibody complexes were washed three times at 4°C with cold lysis buffer and eluted with 2× SDS sample buffer by boiling for 10 min.

### 4.8 Cell‐surface biotinylation

HEK293 WT or SIRT1 KO cells were plated at a density of 1 × 10^5^/mL in 6‐well plates. Cell‐surface biotinylation were carried out as previously described (Bai et al., 2013; Deyts et al., 2019). Briefly, the cells were washed with cold PBS and incubated with sulfo‐NHS‐SS‐biotin (Pierce) at 1 mg/mL for 30 min on ice. Surface biotinylation was stopped by removing that solution and incubating the cells in 10 mM ice‐cold glycine in PBS for 20 min. Cells were rinsed twice in PBS and then lysed in 200 μL modified lysis buffer. A fraction (15%, 30 μL) of the cell lysate was removed to measure total protein concentration and for total input; the remaining 85% (170 μL) of the cell lysate was incubated with 50 μL of 50% streptavidin‐agarose (Pierce) overnight at 4°C. After washing three times the beads were eluted in with 30 μL of 2× sample buffer and boiled. Samples were analyzed by SDS‐PAGE followed by Western blotting using anti‐APP antibody (ab32136, Abcam).

### 4.9 In vitro GST pull‐down

Fusion protein GST‐APP was constructed by inserting the coding region of human APP into pGEX‐5X‐1. The bacterial expression constructs were transformed into BL21‐competent cells (Takara). Cells were induced to protein overexpression by the addition of 1 mM IPTG (isopropyl β‐D‐1‐thiogalactopyranoside) at 30°C for 3 h, and then, they were resuspended in bacterial lysis buffer (50 mM Tris–HCl (pH 8.0), 20% glucose, 10% glycerol, 2 mM MgCl_2_), followed by ultrasonication. The proteins were purified with glutathione sepharose 4B according to the manufacturer's protocol, in which a Flag‐tagged protein synthesized by transcription and translation kit in vitro (Promega, P2221) was added, further incubation for 4 h at 4°C in binding buffer (20 mM Tris–HCl (pH 7.5), 50 mM NaCl, 10% glycerol, 1% NP40). After washing three times with binding buffer, the proteins were eluted with 2× SDS sample buffer by boiling for 10 min. Western blotting was performed to detect bound proteins.

### 4.10 Cell viability assays

Oligomeric Aβ42 solution was freshly prepared according to a published protocol (Brouillette et al., 2012). Briefly, Aβ1‐42 peptides were dissolved in HFIP (Sigma, #105228) to a final concentration of 1 mM, the HFIP treated Aβ peptides were resolved in DMSO and then diluted to a concentration of 113 μM with DMEM/F12 phenol‐red free medium and incubated at 4°C for 24 h. Atomic force microscopy (AFM) analyses further confirmed the Aβ oligomers. Cell viability was analyzed by a Cell Counting Kit‐8 (CCK8; 40203ES76, Yeasen) according to the manufacturer's protocol. SH‐SY5Y cells were (1 × 10^3^/well) cultured in 96‐well plates. Cells were treated with Aβ42 oligomers at 1 day post‐transfection for 70 h. Subsequently, 10 μL CCK‐8 solution was added into each well for an additional 2 h. The viability was detected by using a microplate reader.

### 4.11 LDH toxicity assays

After treatment with Aβ42, supernatants were evaluated by LDH cytotoxicity detection kit (MK401, Takara). One hundred microliters of the cell‐free supernatant were transferred to 96‐well plates and incubated with reaction mixture in the dark for up to 30 min at RT. Then, the absorbance of the samples at 490 nm was measured using a microplate reader. For background control, non‐transfected cultures were used, and high control was measured in the supernatants of cells lysed with 2% Triton X‐100. The percentage of toxicity was calculated as indicated by the manufacturer.

### 4.12 Flow cytometric analysis

To determine cell apoptosis, cells were transfected using indicated vectors for 24 h and treated with Aβ42 for 70 h. Next, cells were harvested with EDTA‐free trypsin, and 1 × 10^5^ cells were stained with FITC Annexin V Apoptosis detection kit I (BD Biosciences, 556547) instructions. The frequency of apoptotic cells was analyzed by flow cytometry analysis (BD Accuri. C6 Plus, BD Biosciences).

### 4.13 Atomic force microscopy (AFM) experiment

The oligomeric Aβ42 sample was plated on freshly cleaved mica substrate and then dried with nitrogen gas. AFM measurements were conducted on a NanoWizard Ultra Speed AFM (JPK) in a QI mode. Image analysis was performed using JPKSPM Data Processing software.

### 4.14 Statistical analysis

All data are presented as mean ± standard error of the mean. Statistical analyses were performed using Student's *t* test for the comparison of two groups; one‐way ANOVA with Tukey post hoc test for the comparison of multiple groups. n represents number of animals or times of repeated experiments. Data analyses were performed using GraphPad Prism 8 (GraphPad Software). *p* < 0.05 was considered significant. *p* values were presented as *p* > 0.05 (ns, not significant), ImageJ software was used to quantify the average fluorescence intensity and the expression of the protein.

## AUTHOR CONTRIBUTIONS

Na Li, Ning Bai, and Liu Cao conceived and designed the experiments. Na Li, Ning Bai, Xiong Zhao, Rong Cheng, Xuan Wu, Bo Jiang, Xiaoman Li, Mingli Xue, Sunrun Cao, and Yanling Feng carried out most of the experiments. Xiong Zhao, Hongde Xu, Qiqiang Guo, Wendong Guo, and Mengtao Ma involved in data acquisition and analysis. Xiaoyu Song, Zhuo Wang, Xiaoyu Zhang, Yu Zou, and Difei Wang provided reagents. Ning Bai and Liu Cao wrote the manuscript.

## FUNDING INFORMATION

This work was supported by the Key Project of the Natural Science Foundation of China (82030091), the Key Project of Liaoning Science Foundation (2022JH6/100100037, 2022JH2/20200034, 2021JH2/10300023), the Natural Science Foundation of China (81902859), The Natural Science Foundation of Liaoning Province of China, Grant/Award Number: LJKZ0735.

## CONFLICT OF INTEREST STATEMENT

The authors declare no competing interests exist.

## Supporting information


**Figure S1.** Expression of SIRT1 and SIRT2 in the brains of APP/PS1 mice.
**Figure S2.** SIRT1 and SIRT2 have opposite effects on cell survival under Aβ42 challenge.
**Figure S3.** SIRT2 overexpression did not change APP locolization in EE.

## Data Availability

The data that support the findings of this study are available from the corresponding authors upon request.
