# Supplementary material for: Cooperative effects of SIRT1 and SIRT2 on APP acetylation
Source: Aging Cell. 2023 Aug 21;22(10):e13967. doi: 10.1111/acel.13967 (PMC10577574; doi:10.1111/acel.13967)
Supplement: Supplementary file 1 — Figure S1. Expression of SIRT1 and SIRT2 in the brains of APP/PS1 mice. Figure S2. SIRT1 and SIRT2 have opposite effects on cell survival under Aβ42 challenge. Figure S3. SIRT2 overexpression did not change APP locolization in EE. [file ACEL-22-e13967-s001.pdf]

Supporting information

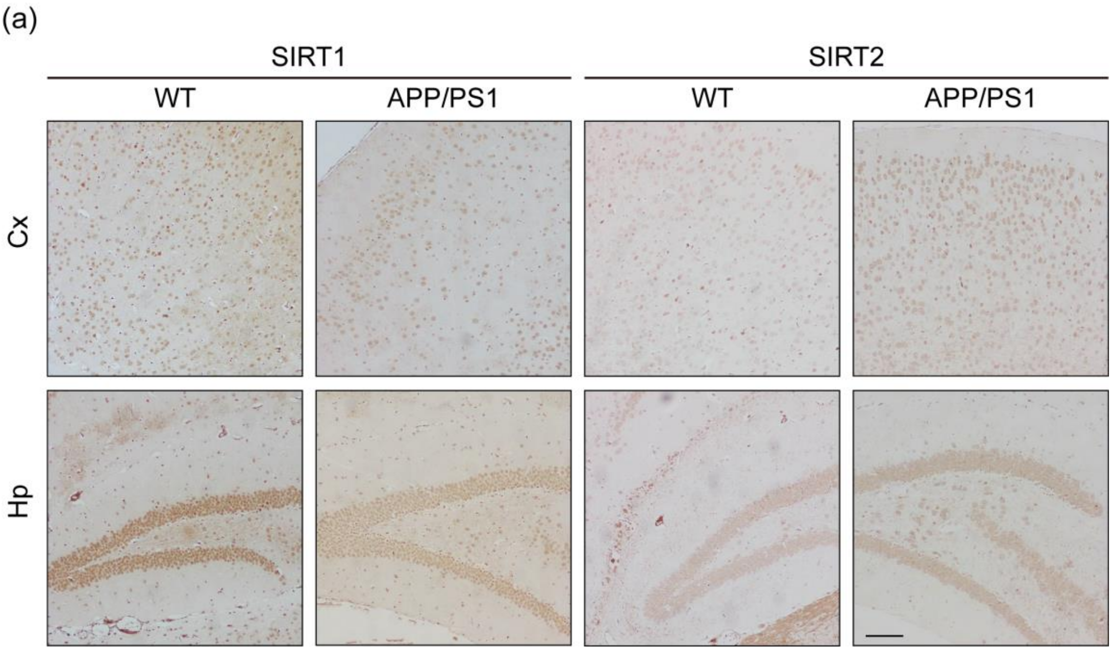

Supplementary Figure S1. Expression of SIRT1 and SIRT2 in the brains of APP/PS1 mice. (a) Representative immunostaining images of SIRT1 and SIRT2 in the frontal cortex (Cx) and Hippocampus (Hp) of WT or APP/PS1 mice (3-month-old). Scale bar: 100 μm.

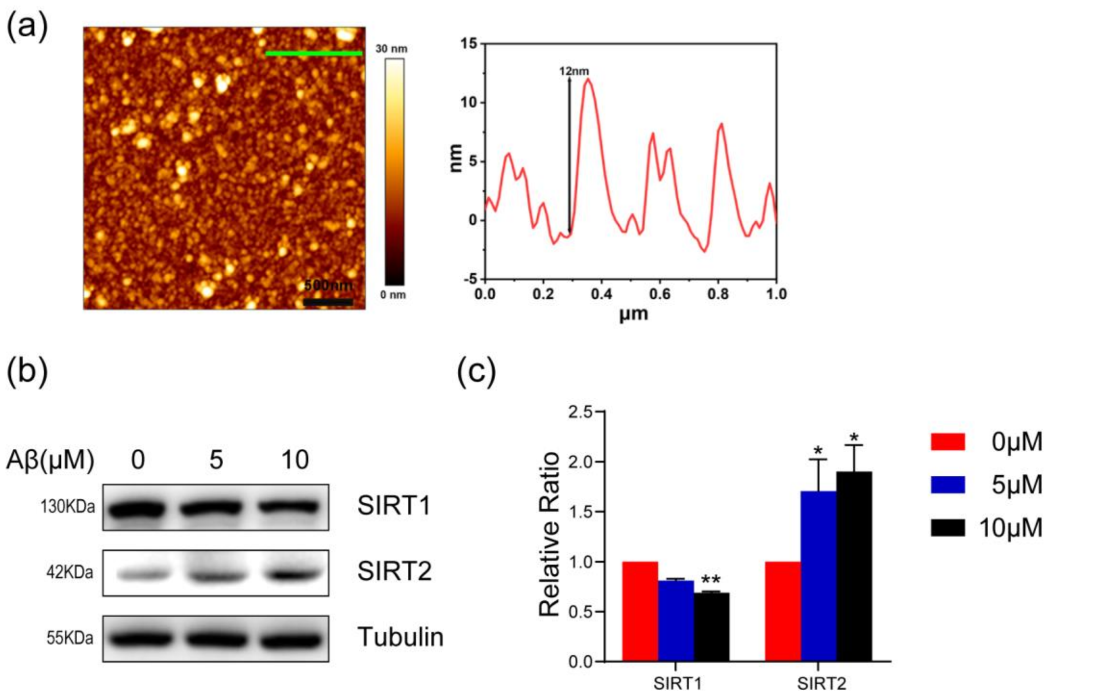

Supplementary Figure S2. SIRT1 and SIRT2 have opposite effects on cell survival under Aβ42 challenge. (a) Atomic force microscopy (AFM) images of Aβ42 oligomers and the corresponding section profile along the green line is shown in the right panel. (b, c) Western blotting and quantification of the protein expression level of SIRT1 and SIRT2 upon Aβ42 stimulus (0, 5, 10 μM) in HT22 cells (n=4). Asterisk depicts p values (* $p < 0.05$, ** $p < 0.01$) as observed by Student's t-test (c), as applicable

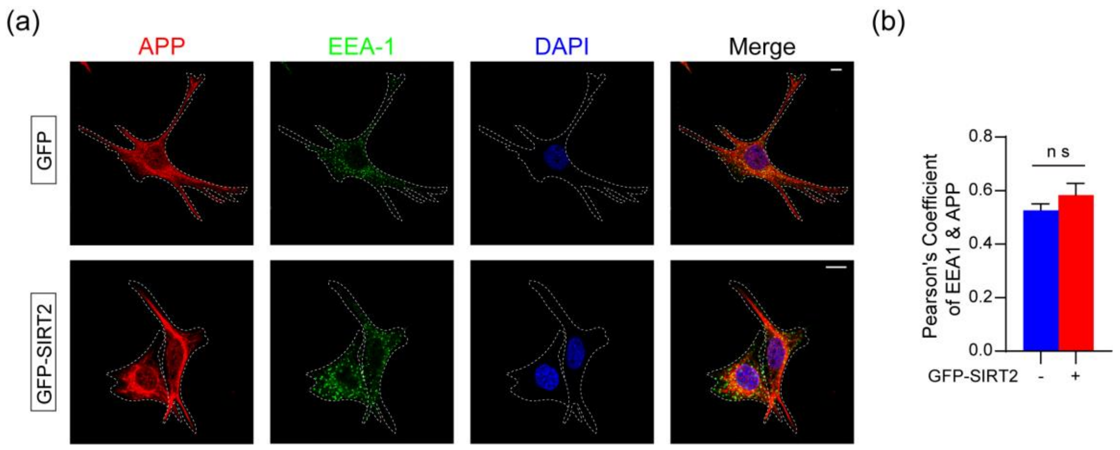

Supplementary Figure S3. SIRT2 overexpression did not change APP locolization in EE. (a) IF of HEK293 cells or HEK293-SIRT1 KO cells using the indicated antibodies. Dashed lines indicate the cell profile (Scale bar= 10 μm). (b) Quantitative analysis of co-localization of APP with EEA1 in the left panel (n=4 fields of vision).
